# "While the wolf is away": the echo of globalization delaying family decisions in intensive care

**DOI:** 10.62675/2965-2774.20240008-en

**Published:** 2024-05-07

**Authors:** Cassiano Teixeira, Diogo Bolsson de Moraes Rocha, Maria Doroti Sousa da Rosa

**Affiliations:** 1 Universidade Federal de Ciências da Saúde de Porto Alegre Internal Medicine Department and Rehabilitations Sciences Porto Alegre RS Brazil Internal Medicine Department and Rehabilitations Sciences, Universidade Federal de Ciências da Saúde de Porto Alegre - Porto Alegre (RS), Brazil.; 2 Hospital Moinhos de Vento Intensive Care Unit Porto Alegre RS Brazil Intensive Care Unit, Hospital Moinhos de Vento - Porto Alegre (RS), Brazil.

Globalization is a complex process that is defined as the "shrinking" of our world through advances in technology and industry; specifically, individuals, peoples, and nations that are very distant from each other are now in contact and may share at least some aspects of a "global" culture.^(1)^ Globalization is multifaceted by nature, affecting society economically, socially, and culturally.^(2)^ Consequently, globalization also impacts health care and medicine. Increases in migration cause individuals and families to be scattered across diverse locations.^(2)^ Career opportunities, academic pursuits, and personal preferences frequently motivate individuals to move away from their family. Concurrently, there is a clear trend toward the aging of the world’s population.^(1)^ The combination of these two phenomena results in complex dynamics between family members who migrate (and who may or may not return, even if only temporarily) and those who stay.^(3)^

Studies on the care of older individuals from a migration perspective are still lacking.^(4)^ One specific aspect of migration that merits attention is its impact on decision-making in critical scenarios. When a parent or other close family member becomes severely ill, swift and well-informed decisions about their treatment are necessary. However, globalization-induced physical separation has the potential to slow the decision-making process, given that family members often reside in different time zones or even on different continents, obstructing communication and coordination. The intricacy of health care choices in the intensive care unit cannot be minimized. Families must discuss matters related to life-preserving treatments, palliative care, and quality of life. These decisions, which are intrinsically difficult, become even more difficult in families separated by migration. In the case of elderly people who remain in their home country, the younger generation that has migrated may be called upon to participate or even lead these discussions and often must deal with feelings of guilt and internal and intrafamilial conflicts.^(1)^

To provide the best care for these patients, an in-depth understanding of the patient’s medical condition and prognosis, along with an awareness of the patient’s desires and values, is needed. Health care professionals and specialist teams who are well versed in critical care often must intervene and assume responsibility for decision-making, particularly in urgent cases. Additionally, the involvement of medical social workers and health care ethicists can be crucial in guiding families through these tough decisions. Consequently, globalization can unintentionally transfer decision-making responsibilities from family members to health care providers. This transition may seem necessary initially, considering the practical challenges caused by distance, but it also raises crucial ethical and emotional questions. Health care professionals are tasked with making decisions that align with the patient's best interests when family members cannot be physically present or actively partake in the decision-making process. While this approach may expedite critical care decisions, it also increases the risk of neglecting the patient's personal values, cultural beliefs, and individual wishes.

Health care teams need to establish clear communication channels with distant family members. Regular updates and consultations with family members are vital to ensure that their input is considered. Hospitals can play a pivotal role in fostering these connections, recognizing the significance of family participation in decision-making. They should take the initiative in designing structures and protocols that support family involvement and joint decision-making. In addition to traditional family meetings and advanced health care directives, superior alternatives such as the use of telehealth technology should be pursued to mitigate the impact of family separation. This approach offers a viable means of communicating and extending care over physical distances, as evidenced during the recent COVID-19 pandemic.^(5)^ Medical social workers play an indispensable role in bridging the gap between families separated by geography and health care providers handling the patients. Their proficiency in counseling, support, and advocacy can be priceless in times of crisis. Medical social workers can help communicate the patient's desires, values, and preferences, even when the family is physically absent. Moreover, cultural competency training is necessary to better understand the varied backgrounds and values of patients and their families, an effort that should begin even before clinical practice.^(6)^ This approach ensures that health care providers respect and accommodate individual beliefs and preferences while improving health care outcomes and satisfaction.^(7)^

In summary, globalization has undeniably reshaped how we live, work, and interact with one another. It has also introduced distinct challenges in health care. The transfer of decision-making responsibilities from family members to health care professionals may be necessary but should be approached with care, focusing on communication, cultural awareness, and the patient's individual values and preferences. Hospitals play a pivotal role in facilitating this process, ensuring that family involvement remains central to decision-making in critical care, even in a globalized world. Collaboration and empathy can be employed to navigate these challenges and ensure that patients' best interests are always foremost.
