# Supplementary material for: "While the wolf is away": the echo of globalization delaying family decisions in intensive care
Source: Crit Care Sci. 2024 May 7;36:e20240008en. doi: 10.62675/2965-2774.20240008-en (PMC11098066; doi:10.62675/2965-2774.20240008-en)
Supplement: Translation

# Translation: "Enquanto seu lobo não vem": o eco da globalização na demora de decisões familiares no cuidado intensivo

**DOI:** 10.62675/2965-2774.20240008-pt

**Authors:** Cassiano Teixeira, Diogo Bolsson de Moraes Rocha, Maria Doroti Sousa da Rosa

**Affiliations:** 1 Porto Alegre RS Brasil Departamento de Medicina Interna e Ciências da Reabilitação, Universidade Federal de Ciências da Saúde de Porto Alegre - Porto Alegre (RS), Brasil.; 2 Porto Alegre RS Brasil Unidade de Terapia Intensiva, Hospital Moinhos de Vento - Porto Alegre (RS), Brasil.

A globalização é um processo complexo, definido como o "encolhimento" do nosso mundo por meio de avanços tecnológicos e industriais; especificamente, indivíduos, povos e nações muito distantes entre si agora estão em contato e podem compartilhar pelo menos alguns aspectos de uma cultura "global".^(1)^ A globalização é multifacetada por natureza, afetando a sociedade economicamente, socialmente e culturalmente.^(2)^ Dessa forma, a globalização também impacta a saúde e a medicina. O aumento da migração promove a dispersão de indivíduos e famílias por diversas localidades.^(2)^ Oportunidades de carreira, aspirações acadêmicas e preferências pessoais frequentemente motivam os indivíduos a se mudarem para longe de suas famílias. Ao mesmo tempo, há uma clara tendência para o envelhecimento da população mundial.^(1)^ A combinação desses dois fenômenos resulta em dinâmicas complexas entre membros da família que migram (e que podem ou não retornar para o seu país de origem, mesmo que apenas temporariamente) e aqueles que ficam.^(3)^

Estudos sobre o cuidado de idosos sob a perspectiva da migração ainda são escassos.^(4)^ Um aspecto específico da migração que merece atenção é seu impacto na tomada de decisões em cenários críticos. Quando um genitor ou outro membro próximo da família fica gravemente doente, decisões rápidas e bem-informadas sobre seu tratamento precisam ser tomadas. No entanto, a separação física causada pela globalização tem o potencial de retardar o processo de tomada de decisão, uma vez que os familiares frequentemente residem em diferentes fusos horários ou até mesmo em diferentes continentes, dificultando a comunicação e a coordenação. A complexidade das decisões referentes ao cuidado na unidade de terapia intensiva não pode ser minimizada. As famílias devem discutir assuntos relacionados a tratamentos de prolongamento da vida, cuidados paliativos e qualidade de vida. Essas decisões, que são intrinsecamente difíceis, tornam-se ainda mais complicadas em famílias separadas pela migração. No caso de idosos que permanecem em seu país de origem, a geração mais jovem que migrou pode ser chamada a participar ou até mesmo liderar essas discussões e frequentemente precisa lidar com sentimentos de culpa e conflitos internos e intrafamiliares.^(1)^

Para fornecer o melhor cuidado para esses pacientes, é necessária uma compreensão aprofundada da condição médica e do prognóstico do paciente, além de ter consciência dos desejos e valores do paciente. Profissionais de saúde e equipes especializadas em terapia intensiva em geral precisam intervir e assumir a responsabilidade pela tomada de decisões, especialmente em casos urgentes. Além disso, o envolvimento de assistentes sociais e especialistas em ética médica pode ser crucial para orientar as famílias nessas decisões difíceis. Consequentemente, a globalização pode, de forma não intencional, transferir as responsabilidades de tomada de decisão dos familiares para os profissionais de saúde. Considerando os desafios práticos causados pela distância, essa transferência pode, em um primeiro momento, parecer necessária. No entanto, levanta questões éticas e emocionais importantes. Os profissionais de saúde são encarregados de tomar decisões que estejam alinhadas com o melhor interesse do paciente quando os familiares não podem estar fisicamente presentes ou participar ativamente do processo de tomada de decisão. Embora essa abordagem possa acelerar as decisões nos cuidados intensivos, ela também aumenta o risco de negligenciar os valores pessoais, as crenças culturais e os desejos individuais do paciente.

As equipes de saúde precisam estabelecer canais de comunicação claros com membros distantes da família. Atualizações e consultas frequentes são essenciais para garantir que a opinião dos familiares seja considerada. Os hospitais podem desempenhar um papel crucial em fomentar essas conexões, reconhecendo a importância da participação da família na tomada de decisão. Estes devem tomar a iniciativa de projetar estruturas e elaborar protocolos que apoiem o envolvimento familiar e a tomada de decisão conjunta. Além das tradicionais reuniões familiares e diretivas antecipadas de vontade, alternativas mais práticas, como o uso de tecnologia de telemedicina, devem ser buscadas para mitigar o impacto da separação familiar. Essa abordagem oferece um meio viável de comunicação e extensão de cuidados perante a distância física, como evidenciado durante a recente pandemia da doença pelo coronavírus 2019 (COVID-19).^(5)^ Assistentes sociais desempenham um papel indispensável na ponte entre famílias geograficamente separadas e profissionais de saúde que cuidam dos pacientes. Sua proficiência em aconselhamento, apoio e advocacia pode ser inestimável em tempos de crise. Os assistentes sociais podem ajudar a comunicar os desejos, os valores e as preferências do paciente, mesmo quando a família está fisicamente ausente. Além disso, a capacitação em competência cultural é essencial para entender melhor as diversas origens e valores dos pacientes e de suas famílias, um esforço que deve começar mesmo antes da prática clínica.^(6)^ Essa abordagem garante que os profissionais de saúde respeitem e acomodem as crenças e preferências individuais, enquanto melhoram os resultados e a satisfação com os cuidados de saúde.^(7)^

Em resumo, a globalização inevitavelmente remodelou a maneira como vivemos, trabalhamos e interagimos uns com os outros. Também introduziu desafios distintos na área da saúde. A transferência de responsabilidades da tomada de decisão da família para os profissionais de saúde pode ser necessária, mas deve ser abordada com cuidado, focando na comunicação, na conscientização cultural e nos valores e preferências individuais do paciente. Os hospitais desempenham papel fundamental na facilitação desse processo, garantindo que o envolvimento familiar permaneça central para a tomada de decisão em cuidados intensivos, mesmo em um mundo globalizado. A colaboração e a empatia podem ser empregadas para navegar por esses desafios e garantir que os melhores interesses dos pacientes estejam sempre em primeiro lugar.
